# The relationship of undernutrition/psychosocial factors and developmental outcomes of children in extreme poverty in Ethiopia

**DOI:** 10.1186/s12887-018-1009-y

**Published:** 2018-02-09

**Authors:** Berhanu Nigussie Worku, Teklu Gemechu Abessa, Mekitie Wondafrash, Marleen Vanvuchelen, Liesbeth Bruckers, Patrick Kolsteren, Marita Granitzer

**Affiliations:** 1 Department of Psychology, Jimma University, Jimma, Ethiopia; 2 Department of Special Needs and Inclusive Education, Jimma University, Jimma, Ethiopia; 3 Department of Population and Family Health, Jimma University, Jimma, Ethiopia; 4 REVAL Rehabilitation Research Centre, Biomedical Research Institute, Faculty of Medicine & Life Sciences, Hasselt University, Hasselt, Belgium; 5 Interuniversity Institute for Biostatistics and Statistical Bioinformatics, Hasselt University, Hasselt, Belgium; 6 Department of Food Safety and Food Quality, Faculty of Bioscience Engineering, Ghent University, Ghent, Belgium

**Keywords:** Undernutrition, Extreme poverty, Psychosocial factors, Developmental outcomes

## Abstract

**Background:**

Extreme poverty is severe deprivation of basic needs and services. Children living in extreme poverty may lack adequate parental care and face increased developmental and health risks. However, there is a paucity of literature on the combined influences of undernutrition and psychosocial factors (such as limited play materials, playground, playtime, interactions of children with their peers and mother-child interaction) on children’s developmental outcomes. The main objective of this study was, therefore, to ascertain the association of developmental outcomes and psychosocial factors after controlling nutritional indices.

**Methods:**

A community-based cross-sectional study design was used to compare the developmental outcomes of extremely poor children (*N* = 819: 420 girls and 399 boys) younger than 5 years versus age-matched reference children (N = 819: 414 girls and 405 boys) in South-West Ethiopia. Using Denver II-Jimma, development in personal-social, language, fine and gross motor skills were assessed, and social-emotional skills were evaluated using the Ages and Stages Questionnaires: Social-Emotional (ASQ: SE). Nutritional status was derived from the anthropometric method. Independent samples t-test was used to detect mean differences in developmental outcomes between extremely poor and reference children. Multiple linear regression analysis was employed to identify nutritional and psychosocial factors associated with the developmental scores of children in extreme poverty.

**Results:**

Children in extreme poverty performed worse in all the developmental domains than the reference children. Among the 819 extremely poor children, 325 (39.7%) were stunted, 135 (16.5%) were underweight and 27 (3.3%) were wasted. The results also disclosed that stunting and underweightness were negatively associated with all the developmental skills. After taking into account the effects of stunting and being underweight on the developmental scores, it was observed that limited play activities, limited child-to-child interactions and mother-child relationships were negatively related mainly to gross motor and language performances of children in extreme poverty.

**Conclusion:**

Undernutrition and psychosocial factors were negatively related to the developmental outcomes, independently, of children living in extreme poverty. Intervention, for these children, should integrate home-based play-assisted developmental stimulation and nutritional rehabilitation.

## Background

Extreme poverty is severe deprivation of nutrition, safe drinking water, sanitation facilities, health care, housing, access to services and protection from violence [1]. Based on data from 89 countries, there were about 385 million children living in extremely poor households in 2013 [2]. The majority of these children in extreme poverty live in South Asia and Sub-Saharan Africa [2, 3] including Ethiopia where many households struggle with famine, chronic drought and malnutrition. Children living in extreme poverty lack adequate care and face increased health risks [4] mainly because poverty is strongly associated with malnutrition, poor sanitation and hygiene, poor maternal education, increased maternal stress and depression, and inadequate stimulation at home [3, 5].

The earlier poverty strikes in the developmental process, the more damaging and long-lasting its effects [6]. Children who are trapped in poverty for a relatively longer period suffer from the worst developmental problems [6] and are less likely to become productive adults [3]. Food insecurity and childhood undernutrition are highly rooted in extreme poverty, and detrimentally affect a child’s cognitive, language, social-emotional and motor development. School attainment, academic performances and social adjustment of children in extreme poverty could also be affected [3, 7].

There is little evidence describing these children’s developmental outcomes and, the nutritional and psychosocial factors associated with them. Only few studies have been conducted to investigate the relationship between poverty and undernutrition. For instance, in a multi-national cohort study in Ethiopia, India, Peru and Vietnam, the association between poverty and childhood undernutrition was explored in children between 6 and 17 months of age, and a few years later when these children were between 4.5 and 5.5 years of age. The study revealed that children living in low-resource contexts had significantly increased probabilities of being stunted and underweight in comparison to children residing in more affluent families [7]. Another study conducted on children of 6–59 months of age in North-West Ethiopia showed that higher monthly family income was inversely associated with stunting [8]. Other studies examined the association between undernutrition and developmental outcomes. A comprehensive review, for example, has shown that poverty and food insecurity could detrimentally affect the development of young children aged from birth to 3 years [9]. For children younger than five living in developing countries, cognitive, motor and social-emotional developmental skills were affected. Early exposure to adversities also compromised their brain development and educational performances [3, 5]. Furthermore, harsh socioeconomic conditions were negatively associated with cognitive performance at 5 years of age [10]. A study conducted on Zanzibari children of 5–19 months old revealed that stunting negatively affected motor and language development [11]. Reduced stunting, better maternal education and stimulation enhanced growth and intellectual development of children in early ages [12].

Stunting and poor psychosocial stimulation were associated with impaired behavioral development [13]. Furthermore, quality parent-child interaction supported development, particularly language and neurocognitive outcomes, of children in low-income families [14]. Play and quality caregiver-child interaction are important for the social, emotional, cognitive, language and motor development of children beginning in early childhood [15]. Nonetheless, children who live in extreme poverty often fail to enjoy their right to play, which consequently affects their development [15].

Even though these studies are essential, it has not yet been explored whether psychosocial factors, after controlling undernutrition, further influence the developmental outcomes of children living in extreme poverty. An investigation of the correlations between these factors and the developmental outcomes could be very useful to design early and need-based interventions.

Within this context, the first objective of this study was to determine the nutritional status and psychosocial factors of Ethiopian children under five living in extreme poverty. The second objective was to compare their developmental performance against age-matched reference peers belonging to families of a middle or higher socio-economic level in Jimma town. As the third and final objective, in the context of nutritional indicators, the influence of psychosocial factors on the developmental outcomes of children in extreme poverty was evaluated.

## Methods

### Study setting

This research was undertaken in Jimma town, South-West Ethiopia. The population in Jimma town was estimated to be 198, 0228 in 2016 [16]. Diverse ethnic and religious groups live together and though many languages are spoken in the town, Amharic and Afan Oromo are the two dominant ones. Because of its multilingual, multicultural, and divergent socio-economic nature, Jimma town reflects, and is representative of most settings within Ethiopia [17].

### Study design

A community-based cross-sectional study was conducted from March 1st to September 2nd, 2013.

### Sampling and study participants

Children in extreme poverty were included in this study if they were (1) between 3 and 61 months of age, (2) living in Jimma town and (3) living in extreme poverty, as validated by the Office of Women’s and Children’s Affairs. However, children (1) with observable physical disabilities which hinder the performance of items in Denver II-Jimma, or (2) who were completely blind or deaf, were excluded. The eligibility of these children for the study was evaluated by pediatricians, mental health professionals, special needs experts and psychologists.

*Sample size estimation and power analysis:* A sample of 672 children per group was required to obtain an 80% power for detection of a difference of 0.07 developmental performance ratio between the extremely poor and reference children when performing a two tailed test at significance level of 0.05. To allow a little more than 20% dropout, 823 children were recruited per group. For the power calculation, the variance in developmental performance ratio scores of 62 children in SOS village-Jimma was used. Their age ranged from 3.5–72 months (mean = 44.6; SD ± 21.2).

The 823 children were randomly selected from 1496 children living in extreme poverty, using a lottery method. The 1496 children were selected and registered by Jimma town’s Women’s and Children Affairs Office. The office selected these extremely poor children using a multidimensional deprivation methodology developed by UNICEF [1]. The generally accepted multidimensional definition of extreme poverty is severe deprivation of nutrition, safe drinking water, sanitation facilities, health care, housing, access to services and protection from violence [1]. Four children were dropped because of incomplete personal data. The remaining 819 children (420 boys and 399 girls) were then enrolled for the study. All their caregivers were also requested to provide the necessary information.

The developmental outcomes of these children in extreme poverty were compared to 819 (405 boys and 414 girls) age-matched reference children. They were randomly selected using a lottery method from 1, 597 children. Children in this reference group live with families of a middle or higher socio-economic level and their nutritional status varied between the Z-scores of -2SD and +2SD, implying that they were not malnourished according to WHO Child Growth Standards [18]. Both children in extreme poverty and the reference group lived in Jimma town and were assessed in the same time period.

### Outcomes, measurements and instruments

#### Developmental outcomes

The developmental performance of each child was assessed using the Denver II-Jimma [17], a version of the Denver II [19], adapted to the sociocultural context of children under six living in the Jimma zone of Ethiopia. Denver II-Jimma has an excellent inter-rater and test-retest reliability on the majority of the items in the test [17]. It consists of 125 items: 25 personal-social, 29 fine motor, 39 language and 32 gross motor. Most of these items require children to perform, and a few are based on their parents’ report. On average, testing with the Denver II-Jimma took around 20 min. For each child, the performance ratio for each developmental domain was calculated. Performance ratio simply refers to the ratio of the total number of items passed to the expected number of items a child should pass taking into account the child’s age [17]. Children performing lower than what is expected for their age, have a performance ratio below one.

To test the social-emotional development of the children, ASQ: SE questionnaires were used. These are parent-report questionnaires developed to identify children whose social and emotional competences might differ from what is expected [20]. ASQ: SE is recommended for its high rate of detection of social-emotional problems among young children [21]. It has high test-retest reliability [20]. The questionnaires were culturally adapted to the study context and translated into both local languages. An ASQ: SE questionnaire only took about 10–15 min to complete for a caregiver. The child’s total score was calculated by adding up the points of all items on the questionnaire. A higher total score indicates more social-emotional problems.

#### Nutritional status

To characterize the nutritional status, anthropometric assessment was done following WHO guidelines [18]. The child’s weight was measured using a calibrated electronic weighing scale (SECA Uniscale, Hamburg, Germany). For children under 2 years, the length was measured using a length-measuring mat on a flat table (SECA 210, Hamburg, Germany). The height of a child above 2 years was measured by using a Seca Road Rod 214 portable Stadiometer. Age was recorded from birth certificates or immunization cards. If reliable documents for age estimation were absent, local events calendars were used to help the mother or caregiver estimate the approximate age of the child.

#### Demographic and psychosocial characteristics

A structured questionnaire was used to collect the demographic and psychosocial characteristics of the participants. Some of the variables measured were maternal age, education and occupation, age, sex and birth order of the child, monthly income of the household, child feeding condition, number of persons living under the same roof with the child, availability of play materials and playground, time spent on play by the child, mother-child interaction, and frequency of a child’s interaction with other children.

### Testing procedure

We obtained ethical approval from The Ethical Review Board of Jimma University, Ethiopia (RPGC/36/2013, dated 13/02/2013). We also obtained written informed consents from the children’s parents. Measurements and testing were performed by four trained nurses, who spoke both Afan Oromo and Amharic languages. They were trained for 1 month by a child development expert and a nutritionist on the theoretical and practical aspects of overall child development, care, nutrition, anthropometric methods, Denver II-Jimma, the structured questionnaire and ASQ: SE. To reduce possible biases, the testers were blinded; they did not know to which group a child belonged. The testers assessed children in both groups and the assessments were performed at the children’s homes while their caregivers were present. Prior to testing, the tester created a relaxing environment with the child and its respective caregiver. Regarding the assessment order, a questionnaire, together with ASQ: SE, was administered first; the Denver II-Jimma next and finally, the anthropometric measurements. The assessment time for a child took about an hour.

### Statistical analysis

To guarantee data quality, double data entry was done into EpiData. The data were then exported to SPSS version 22 and analyzed.

The anthropometric measures were converted into Z-scores of Weight-for-Age (WAZ), Height/Length-for age (HAZ/LAZ), and Weight-for-Height/Length (WHZ/WLZ) using WHO Anthro and Anthro plus software [22]. Based on the WHO reference standard, underweight, wasting and stunting were defined as WAZ, WHZ/WLZ and HAZ/LAZ below -2SD respectively. Z-scores between -3SD and -2SD represent moderate undernutrition; whereas, the Z-scores below -3SD signify severe undernutrition. ‘Not-malnourished’ children are the children whose Z-scores lay between -2SD and +2SD for the three nutritional indices.

The data indicated a prevalence of stunting, wasting and underweight of the children in extreme poverty. To compare the psychosocial conditions of children in extreme poverty and the reference group, chi-square tests (χ^2^) were employed. Independent samples t-test was used to compare the developmental outcomes of children in extreme poverty and the reference group. To determine the association between the developmental scores and the nutritional/psychosocial indicators, for children in extreme poverty, multiple linear regression analyses were used for each developmental score (personal-social, fine motor, language and gross motor) separately. Two-stage approach was carried out. A regression model with the two nutritional indicators (stunting and underweight) as independent variables was fitted. Both nutritional indicators were significantly associated with each developmental score. Next, the demographic and psychosocial factors (child’s sex, birth order, feeding condition, maternal age, education, occupation, monthly family income, family size, availability of play materials, availability of playground, play time, child-to-child interaction and mother-child relationship) were added to the regression model containing the statistically significant nutritional indices. Finally, a parsimonious model was obtained by means of a stepwise selection. The significance level was set at 0.05 and all tests were two-sided.

## Results

### Nutritional and psychosocial characteristics of children in extreme poverty

Regarding the type and degree of undernutrition of children in extreme poverty, 213 (26%), 99 (12.1%) and 19 (2.3%) were moderately stunted, underweight and wasted, respectively; whereas, 112 (13.7%), 36 (4.4%) and 8 (1%) children were severely stunted, underweight and wasted, respectively. Within the reference group, no child was malnourished.

Psychosocial factors such as mother-child interaction, availability of play materials and playground were very limited for children in extreme poverty, unlike for children in the reference group. Nevertheless, the children spent most of their time playing with whatever was accessible to them, and wherever possible, alone or with their peers. The details are presented in Table 1. Some psychosocial and demographic factors such as maternal age, maternal occupation and education level, family size, income, sex and birth order of the child were not included in Table 1, because they showed less meaningful contributions to the developmental outcomes of children in extreme poverty.

**Table 1:** Nutritional status and psychosocial factors of extremely poor and reference children

Variables	Extreme poverty	Reference	χ^2^	*P*-value
	(*n* = 819)	(*n* = 819)		
Nutritional status				
Moderately stunted	213 (26.0%)	–		
Severely stunted	112 (13.7%)	–		
Moderately wasted	19 (3.3%)	–		
Severely wasted	8 (1.0%)	–		
Moderately underweight	99 (12.1%)	–		
Severely underweight	36 (4.4%)	–		
Psychosocial factors				
Child-child interaction (No)	413 (50.4%)	225 (27.5%)	90.7	< 0.001
Mother-child interaction (No)	525 (64.1%)	307 (37.5%)	116.1	< 0.001
Availability of play materials (No)	652 (79.6%)	344 (42.0%)	243.0	< 0.001
Availability of playground (No)	554 (67.6%)	272 (33.2%)	194.2	< 0.001
Play time (No)	193 (23.6%)	135 (16.5%)	12.8	< 0.001
Sex-child (girls)	420 (51.3%)	414 (50.5%)	0.6	= 0.459
Age-child [mean(SD)]	30.40 (15.83)	30.76 (15.83)	0.5	= 0.641

Note. χ^2^ = Chi-square test statistic, P-value = level of significance. Child-child interaction refers to the frequency of a child’s interaction with other children. For the psychosocial factors, we obtained binary (Yes or No) responses. *P*-value of 0.000 was reported as < 0.001. For the children’s age differences, we used t-test statistic. Children’s age ranged from 4.80 to 60.16 months

### Developmental outcomes of extremely poor and reference children

Children living in extreme poverty performed less well in all the four developmental domains of Denver II- Jimma compared to children from the reference group. They also performed worse on social-emotional development indicated by the higher scores (Table 2).

**Table 2:** Developmental outcomes of extremely poor and reference children

Developmental outcomes	Group	Mean	SD	t	Df	*P*-value
PR Personal-social	Extr. poor	.99	.14			< 0.001
	Reference	1.05	.18	7.28	1636	
PR Fine motor	Extr. poor	1.02	.10			< 0.001
	Reference	1.07	.12	7.78	1636	
PR Language	Extr. poor	.96	.13			< 0.001
	Reference	1.03	.14	9.60	1636	
PR Gross motor Social-emotional	Extr. poor	1.04	.11			< 0.001
	Reference Extr. Poor Reference	1.07 67.87 48.68	.10 27.52 27.15	6.32 −14.20	1636 1636	< 0.001

*Note. PR* Performance Ratio, *SD* Standard Deviation, *t* Independent samples t-test

statistic, *Df* Degree of freedom, *Extr*. Extremely, *P*-value level of significance

### Developmental outcomes and undernutrition among children in extreme poverty

To test for the statistical significance of the relation between nutritional indices and developmental outcomes, stunting and underweight were entered into a regression model as independent variables. Stunting was negatively related to personal-social (β = −.077, t (816) = − 2.151, *p* = 0.032), fine motor (β = −.123, t(816) = − 3.448, *p* < 0.001), language (β = −.178, t(816) = − 5.030, *p* < 0.001), and gross motor development (β = −.212, t(816) = − 6.175, *p* < 0.001); and so was underweight to personal- social (β = −.152, t (816) = − 4.260, *p* < 0.001), fine motor (β = −.162, t(816) = − 4.569, *p* = 0.006), language (β = −.157, t(816) = − 4.443, *p* < 0.001), and gross motor development (β = −.283, t(816) = − 8.247, p <  0.001). Wasting, as an independent variable, was not entered into the regression model because of the small number of wasted children.

### Developmental outcomes and psychosocial factors among children in extreme poverty

**Fig. 1:**
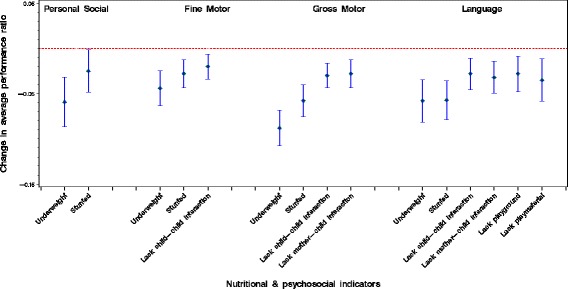
Change in average developmental outcomes versus nutritional/psychosocial indicators

Change in average developmental performance ratio or outcomes versus nutritional/psychosocial indicators are displayed in Fig. 1.

To observe the contribution of psychosocial factors, on top of the nutritional indicators, to personal-social, fine motor, language and gross motor development, the psychosocial factors were added to the regression model containing the nutritional indices. No additional contributions of psychosocial factors to personal-social development were seen. However, for the other developmental outcomes, some psychosocial factors contributed on top of the nutritional indices. For fine motor development, limited availability of play corner (β = −.092, t(815) = − 2.682, *p* = 0.007) was negatively associated; for language development, limited child-to-child interaction (β = −.106, t(812) = − 3.094, *p* = 0.002), reduced mother-child interaction (β = −.115, t(812) = − 3.345, *p* <  0.001), limited availability of play materials (β = −.108, t(812) = − 2.982, *p* = 0.003) and limited play corner (β = −.098, t(812) = − 2.688, p = 0.007) were negatively correlated; and for gross motor development, limited child-to-child interaction (β = −.130, t(814) = − 3.947, *p* < 0.001) and limited mother-child interaction (β = −.120, t(814) = − 3.649, *p* < 0.001) were negatively related. From these findings, it is clear that the psychosocial factors were negatively related mainly to gross motor and language performances.

## Discussion

The main objective of this study was to determine the relationship between the developmental outcomes and psychosocial factors after controlling nutritional indices. This study has also described the nutritional status and psychosocial factors of children in extreme poverty. The developmental outcomes of these children were also compared against the reference children.

Within children in extreme poverty, stunting, wasting and underweightness problems were observed. Stunting or chronic undernutrition was the most dominant type. In Ethiopia, children under five residing in extreme poverty had high odds of being stunted [23]. Stunting was common in low resource setting [24], particularly in Sub-Saharan Africa [25]. One of the possible explanations could be that stunting, especially in the context of extreme poverty, is helical and is highly related to economic capacity. For example, parents who live in an extremely poor environment and had experienced chronic malnutrition themselves during childhood are most likely to have stunted children. If this condition continues, it may create an intergenerational cycle of extreme poverty which is hard to break [13, 26]. Another explanation is that stunting is associated with poor diets of low diversity, poor water supply, poor sanitation and hygiene, and chronic illness [27].

Children in extreme poverty performed more poorly than the reference children in all the developmental domains. A similar performance difference for children ranging from 6 to 42 months, especially in language, was reported in prior research [28]. One of the reasons might be that food insecurity has been indirectly linked to delayed development [9]. Micronutrient deficiencies, food insecurity, infectious disease and parenting stress are factors associated with extreme poverty, and thus, with developmental delays [29, 30]. Moreover, the differences in the developmental outcomes between the two groups might also have its origins in adverse early experiences and the socioeconomic inequality of children in extreme poverty [5]. For example, neighborhood economic disadvantage was indirectly associated with child development [31].

Stunting or chronic malnutrition was negatively associated with all four Denver II developmental domains. A previous study conducted in the rural area of the same region reported that stunting was a risk factor for lower overall developmental scores for children aged 3–24 months [32]. Such an effect on development may be difficult to reverse since, for instance, 11–12 year old children who had recovered from early childhood stunting still showed significantly poorer fine motor skills, even though these children received psychosocial stimulation and nutritional supplementation when they were between the ages of 9 to 24 months [33]. A comprehensive review on studies conducted in low- and middle-income countries has revealed that stunting was associated with delayed development for children less than 2 years of age, and that the underlying causes of stunting may also have direct effects on cognitive and motor development [34]. The study, conducted in Pakistan, also revealed that stunting was negatively related to gross motor development during infancy [35]. This might be because extreme poverty is the major contributor to undernutrition, which becomes a serious hazard to child development [36]. Another explanation is that a lack of nutrients can have long-term effects on the children’s brain structure and on their development [37]. Stunted growth during early childhood is associated with poor development of, especially, language and motor skills [38]. This is mainly because it is related to loss of physical growth potential and, reduced neurodevelopmental and cognitive function [39]. In the context of extreme poverty, it is expected that breast-feeding and complementary feeding frequencies are low, dietary diversity is minimal, and infectious disease morbidities are high. These nutrition-related factors had a significant association with child development, particularly motor and language skills [40].

Our study also showed that the children lacked adequate stimulation, mother-child interaction, play materials and playground. This is expected in the context of extremely resource-limited areas such as this study site [14, 15]. Children who live in extreme poverty often face socioeconomic hindrances that hamper their opportunity to interact with the immediate environment [15]. Consequently, these limited interactions may affect their later self-regulation and functional skills [41].

On top of the nutritional problems, psychosocial factors such as limited play activities, mother-child interaction, and child-to-child interaction were negatively associated with the developmental outcomes of children in extreme poverty. Experiencing adverse psychosocial conditions during childhood may negatively affect particularly the language development and behavior of the exposed children [42]. The crucial role of, for instance, active and free play for language development was stated previously [15]. Such availability of indoor-outdoor play spaces which engage young children could also facilitate the overall development process [43], not to mention the importance of the language input by mothers at home for children’s language development [44]. Other studies have disclosed that the mother-child relationship was significantly associated with language development [45]. On the contrary, if the relationship is negative: for example, repeated rejection, inconsistency in emotion and carelessness on the part of primary caregiver towards the child, it could be a threat for the child’s developmental outcomes [46]. Poor quality of mother-child interaction significantly affected the development of a child in the early years of life [47]. Children experiencing developmental delays were those whose mothers were less responsive and provided less cognitive stimulation [47]. Young children’s exposure to a persistently chaotic household was highly associated with poorer language development [48]. This was the case for our study children who were living in extremely low wealth communities. Moreover, in our study, child-to-child interaction or peer relation was significantly related to gross motor development. This finding is consistent with the study that revealed gross motor skills of children to be related to their peer relation [49]. This is because children mostly interact through different play activities that may involve large muscles. In the context of extreme poverty, these activities are limited.

In this study, variables such as household income, family size, maternal occupation and maternal education were not significantly associated with any of the developmental domains. Among several possible explanations, one is the homogeneous nature of the target population in terms of the aforementioned variables. The majority of the families lived in poorly constructed houses with no pipe water, electricity, or a television or radio receiver. They depended on scant income, which could not adequately feed their large family size. Some of the mothers did small things such as selling seasonal vegetables and fruits on the street and fed their families with the extra money they earned; the rest were housemaids. The families shared a similar context and the differences in the variables were not large enough to explain the variations in the developmental outcomes.

This study is not without limitations. Denver II-Jimma is a screening test and may share the limitations, for example limited specificity, of Denver II [50]. To exclude children from the present study, the experts did not use standardized tools. As a result, there might be a number of children with problems that were not excluded. Some factors, which could significantly be associated with developmental and nutritional outcomes of the children, were not included in this study. These factors include paternal education, age of initiation of complementary feeding, family planning methods used, poor child feeding patterns and breast-feeding practices [51].

For future research, it would be interesting to link the problems in developmental performance due to malnutrition to the status of a child’s brain by functional MRI (Magnetic Resonance Imaging). Nutritional deficiencies have been shown to be associated with neurological changes [52].

## Conclusion

Undernutrition was negatively associated with all the developmental performances of the children in extreme poverty. In addition, psychosocial factors such as reduced play activities, child-child interaction and mother-child relationship were negatively related to, in particular, the gross motor and language development of these children. Interventions, therefore, ought to consider integrated home-based play-assisted developmental stimulation and nutritional rehabilitation.

## Abbreviations

HAZ: Z-score of Height-for-Age
LAZ: Z-score of Length-for-Age
WAZ: Z-score Weight-for-Age
WHZ: Z-score of Weight-for-Height
WLZ: Z-score of Weight-for-Length
